# Antimalarial Activity of *Nigella sativa* L. Seed Extracts and Selection of Resistance in *Plasmodium berghei* ANKA in a Mouse Model

**DOI:** 10.1155/2021/6165950

**Published:** 2021-02-03

**Authors:** Rahma Udu, Job Oyweri, Jeremiah Gathirwa

**Affiliations:** ^1^Department of Pure and Applied Sciences, School of Applied & Health Sciences, Technical University of Mombasa, P.O. Box 90420–80100, Mombasa, Kenya; ^2^Centre for Traditional Medicine and Drug Research, Kenya Medical Research Institute, P.O. Box 54840-0002, Nairobi, Kenya

## Abstract

**Background:**

Chemotherapy plays a crucial role in malaria control. However, the main obstacle to treatment has been the rise of parasite resistance to most antimalarial drugs. Artemisinin-based combination therapies (ACTs) remain the most effective antimalarial medicines available today. However, malaria parasite tolerance to ACTs is now increasingly prevalent especially in Southeast Asia presenting the danger of the spread of ACTs resistance to other parts of the world. Consequently, this creates the need for alternative effective antimalarials. Therefore, this study sought out to determine antimalarial potential, safety, and resistance development of the extracts in a mouse model.

**Method:**

Methanolic and ethyl acetate extracts were obtained by solvent extraction. The extracts were assayed for acute toxicity *in vivo*. Additionally, the two extracts were evaluated for antimalarial activity *in vivo* against *Plasmodium berghei* ANKA strain by the 4-day suppressive test at 500, 250, and 125 mg/kg/day. Packed cell volume was evaluated to determine anemia manifestation. Finally, continuous drug pressure experiment at 500 mg/kg and DNA amplification via PCR were conducted. The amplicons underwent through Sanger sequencing.

**Results:**

There was no toxicity realized in the animals at 2000 mg/kg. Importantly, high parasitemia suppression of 75.52% and 75.30% using a dose of 500 mg/kg of methanolic and ethyl acetate extracts, respectively, was noted. The extracts were able to reverse packed cell volume reduction. *Nigella sativa*-resistant phenotype was selected as delayed parasite clearance. However, there was no change in the nucleotide sequences of *Pb*MDR1 and *Pb*CRT genes.

**Conclusion:**

The results provide room for future exploitation of the plant as an antimalarial.

## 1. Introduction

The fight against malaria in the majority of the developing nations especially in sub-Saharan Africa remains worrisome [1]. The years 2010, 2016, and 2017 recorded 239 million, 217 million, and 219 million malaria cases, respectively. Malaria claimed 607,000 lives in 2010. Subsequently, 2016 and 2017 saw 451,000 and 435,000 people die from the disease [2]. Antimalarial medications have fairly reduced malaria deaths and transmissibility [3]. The World Health Organization recommends the use of Artemisinin Combination Therapy (ACT) to overcome drug resistance. Accordingly, a short-half-life artemisinin derivative clears parasites swiftly using dissimilar way of action from that of the long-acting parent drug that eliminates persistent parasites [4]. Presently, artemether lumefantrine (AL) combination is the first line medication for uncomplicated malaria in Kenya. Despite the malaria reduction ability of ACTs, cases of resistance in the Thailand-Cambodia border, Southeast Asia, have been noted. The resistant strain may spread to Africa and other parts of the world. This calls for new effective intervention approaches including the use of medicinal plants [5–7]. Quinolones and artemisinin derivatives have been established from traditional herbal plants [8].

Resistance development is prominently influenced by resistant mutants within distinct hosts. Drug resistance selection in animal model permits genetic and physiological manipulations. The serial technique (ST) that employs drug dose increment per passage has lately been used to achieve drug pressure. Additionally, the 2% relapse technique (2% RT) that utilizes a high sole drug dose in each passage has been used [9]. However, Siregar et al. [10] have achieved the selection of atovaquone-resistant *P. berghei* strains from incomplete cure runs with a constant therapeutic dose, which explains cure failure in human.

An estimate of 122 modern allopathic drugs has been established from about 94 plant species via ethnobotanical leads [11]. Folk medicinal plants are alleged to be safe. Additionally, they are locally available and efficient and have a friendly way of action [12]. However, more studies are necessary to validate these claims [13]. Asia, Europe, and Africa including India, South Europe, and the African coastlines, respectively, have enjoyed the medicinal value of *N. sativa* [14, 15]. Antioxidant, antidiabetic, anticancer, anti-inflammatory, and antiparasitic effects of the plant have been documented [16–18]. Importantly, studies in Middle East confirm its antiparasitic activity with little or no report on malaria [19]. However, few reports show the antimalarial effects of ethanolic, aqueous, and chloroform extracts of *Nigella sativa* [20]. Elsewhere, Ashcroft et al. [21] have documented methanolic and aqueous extracts' activity of the plant against *P. berghei* (nk 65) infection. Traditionally, the natives in the Coastal Province of Kenya comprising Arabs, Asians, and Bantus use the seeds as a concoction boiled in water [22, 23]. They have normalized the idea of taking the boiled seeds after meals and it has proved to be a promising remedy against malaria associated symptoms including chills, headache, nausea, and lack of appetite, among others. It is in this light that the study sought to find out whether the *Nigella sativa* seeds truly treat *Plasmodia* parasites or the symptoms linked with malaria.

*Plasmodium berghei* lines have been used to determine overexpression of mdr1 gene in mefloquine, which is associated with resistance in *P. falciparum* [24]. The study overlooked the *in vitro* assay of *P. falciparum* due to its difficulty of resistance establishment [25]. The study is based on one postulate that continuous exposure of the malaria parasite to similar test drug concentrations in different animal hosts challenges the parasite to develop ways of overcoming the effect of the test drug.

We principally determined qualitative phytochemical screening of the extracts and antimalarial activity *in vivo*. We determined packed cell volume (PCV) in relation to anemia. Additionally, we selected stable *N. sativa*-resistant strains by continuous drug pressure *in vivo* and amplified *Pb*crt and *Pb*mdr1 genes. Finally, amplicons were sequenced and analysis of sequence variation in *Pb*crt and *Pb*mdr1 was done.

## 2. Materials and Methods

### 2.1. Plant Collection, Authentication, and Extraction

One kilogram of *N. sativa* seed samples was bought from Mwembe Tayari market, Mombasa, and packed in plastic bags (voucher specimen no. PAS0024). The seeds were of Saudi Arabian origin. A taxonomist from KEMRI and indigenous Mombasa residents who traditionally use the plant helped in positive identification. The seed was moved to KEMRI Centre for Traditional Medicine and Drug Research, Nairobi, for handling. Air-dried seeds were ground using an electric laboratory mill (Christy and Norris Ltd., Chelmsford, England). Methanol and ethyl acetate solvents (600 ml) were used to extract 400 g of the ground material. Concisely, 400 g of the ground material was extracted using 600 ml of the respective solvents by maceration at room temperature for 48 h. The soaked plant material was filtered and concentrated to dryness under reduced pressure using rotary evaporator. The obtained crude extracts were further dried in a vacuum drier to get rid of any likely traces of solvents and later stored at −20°C until use.

### 2.2. Animal Ethical Considerations, Animals Used, and Parasite Inoculation

In order to conduct animal experiments, the study obtained permission from KEMRI Animal Care and Use Committee (KEMRI/ACUC/01.07.19). Animal care and use guidelines were provided by KEMRI animal house personnel. The animals were handled in a humane manner following the internationally accepted principles. Gauge 23 needles were used to carry out IP injection. At the end of the experiments, mice were sacrificed in a chamber of carbon (iv) oxide. The carcass was finally incinerated.

Male Swiss albino mice, aged 6 weeks old, weighing 20 ± 2 g, raised in the KEMRI animal facility Nairobi, were used as subjects in all the *in vivo* experiments. The subject animals were weighed using sensitive electronic balance. The housing and feeding conditions were maintained as per the recommended KEMRI standards. Briefly, characteristic microloan type II cages, well branded with experimental details, accommodated five test animals per cage in air-conditioned rooms at 20°C room temperature. Relative humidity was maintained at 60–70%. They were accordingly nurtured with commercial rodent feed and water *ad libitum*. Chloroquine-sensitive *P. berghei* (ANKA strain) parasites were used to confer malaria in mice. Mice previously infected with *P. berghei* were used as donors and the parasites were subsequently kept alive by continuous serial intraperitoneal (IP) passage of blood.

### 2.3. *In Vivo* Acute Toxicity

The acute toxicity study was conducted according to previous research with few modifications [26, 27]. Concisely, five mice per cage were adapted to laboratory conditions for 3 days. Four groups of mice were starved of food but served with clean water ad libitum for 24 h before oral administration of different doses of the test extracts. The test groups received a single dose of 1000, 1500, and 2000 mg/kg of body weight per extract. The negative group of animals received 0.2 ml of the vehicle. In this study, physical changes including shrunk body or/and limb, reduced motor activity, abnormalities in breathing, struggling, dizziness, lack of appetite, rough fur, and spasms in both rear legs and mortality within 24 h were inspected. Furthermore, similar parameters were checked for the subsequent 14 days.

### 2.4. Infecting and Dosing of Animals

Blood was taken from a donor mouse with 30% parasitemia and diluted in physiological saline to 5 × 10^7^ parasitized erythrocytes per ml. Swiss albino mice (male) weighing 20 ± 2 g were infected with 0.2 ml blood (2 × 10^7^*P. berghei* parasitized erythrocytes) intraperitoneally and randomly divided into three test groups and two control groups of 5. 5 mg/kg chloroquine was used as a standard drug and normal saline as a negative control [8].

#### 2.4.1. Four-Day Parasite Suppression Test

Peters' [28] protocol with slight amendments was exploited to complete the four-day parasite suppression test. *P. berghei* strain ANKA infected blood from the donor mouse was mixed with 1% (w/v) heparin. The infected blood was diluted in physiological saline to roughly 10^8^ parasitized erythrocytes per ml. The test animals were intraperitoneally injected with 0.2 ml and indiscriminately grouped into fives. The experimental groups were orally treated with 0.2 ml single dose of 125, 250, and 500 mg/kg of the test sample, 2 h after infection (D0) [29]. The negative and positive control groups were treated with 0.2 ml of the vehicle and 5 mg/kg of CQ, respectively. The mice were successively treated orally for 3 days (D1, D2, and D3) with corresponding doses. Thin blood smears were taken on the fifth day (D4) from tail snips, fixed in methanol, and stained with 10% Giemsa stain [30]. Parasitemia suppression was determined as per Tona et al.'s [27] formula:

(1) $$\begin{array}{c} \text{PS}=\frac{A-B}{A}\times 100, \end{array}$$

where *A* is mean parasitemia in the negative control group on day 4 and *B* is parasitemia in the test group.

The mean survival time (days) for each group was determined over a period of 30 days after infection.

### 2.5. Determination of Packed Cell Volume

A keenly adjusted protocol highlighted by Mekonnen [31] was used to determine packed cell volume (PCV) in the assay. Packed cell volume aids evaluate the efficacy of the test extracts in inhibiting hemolysis consequential from cumulative parasitemia linked to malaria. Concisely, blood was collected from the tail of each mouse in heparinized microhematocrit capillary tubes by filling three-quarters of its volume. The tubes were sealed by sealant and placed in a microhematocrit centrifuge with the sealed ends outwards. The blood was centrifuged at 12,000 rpm for 10 min. The PCV of each mouse was measured before infection and on day 4 after treatment using the following formula [31]:

(2) $$\begin{array}{c} \text{PCV}=\frac{\text{volume of erythrocytes in a given volume of blood}}{\text{total blood volume}}\times 100. \end{array}$$

### 2.6. Determination of Level of Parasitemia

Thin tail blood smears of each mouse were made for the 4-day suppressive test. After fixation with absolute methanol, the slides were stained with 10% Giemsa at pH 7.2 for 15 min. Finally, the slides were gently washed with distilled water and air-dried at room temperature. They were microscopically viewed under 100x magnification power using Olympus microscope oil immersion. The percentage parasitemia was calculated using the following formula [32]:

(3) $$\begin{array}{c} \%\text{parasitemia}=\frac{\text{number of parasitized RBCs}}{\text{number of total RBCs}}\times 100. \end{array}$$

### 2.7. Determination of Mean Survival Time

A 30-day follow-up time was implemented to monitor death amongst experimental animals in respective groups. Incidences of death were recorded regardless the group. Mean survival time (MST) of mice of each group was determined using the following formula:

(4) $$\begin{array}{c} \text{MST}=\frac{\text{sum of survival time of all mice in a group}\left(\text{days}\right)}{\text{total number of mice in that group}}. \end{array}$$

### 2.8. Exerting Drug Selection Pressure and Assessing the Level of Resistance

The experiment was run with minor changes as per Kangethe's [33] protocol. Concisely, intraperitoneal inoculation of 0.2 ml of parasitized red blood cells was carried out on 5 mice per dose group on day 0 (D0). The infected mice were left untreated until parasitemia rose to 8%. As a result, 500 mg/kg dose of test extracts was initiated. Treatment was carried out as described earlier on in the four-day suppressive test. Parasitemia was monitored until it hit above 10% which could cause animal death. The mice were then selected for passaging of parasitized red blood cells to the next fresh group of 5 mice per test sample. This was carried out for the next 15 cycles, with the same test drug concentration. Resistance level was determined after every 5 cycles of passaging (approximately one month). The ED_50_ and ED_90_ values were obtained by plotting a linear dose-response curve. The standard four-day suppressive test allowed evaluation of “index of resistance,” RSI_50_ and RSI_90_ (well known as the ratio of the ED_50_ and ED_90_ of the resistant line to that of the sensitive, parent line).

The RSI_50_ and RSI_90_ values were categorized into four classifications, based on past research work [34]: (1) RSI_50/90_ = 1.0, sensitive; (2) RSI_50/90_ = 1.01–10.0, slight resistance; (3) RSI_50/90_ = 10.01–100.0, moderate resistance; and (4) RSI_50/90_ > 100.0, high resistance.

### 2.9. Cryopreservation of *P. berghei* ANKA Parasitized Red Blood Cells

0.5 ml of 2–8% *P. berghei* ANKA PRBCs from cardiac puncture was collected in 0.05 ml heparin. The blood was mildly mixed with 0.5 ml of glycerol/physiological saline solution (comprising 20% glycerol; v/v) in cryotubes. The tubes were then stored at 4^o^C for 10 minutes and transferred to −80°C as illustrated by Muthui [35].

### 2.10. Primers for Molecular Work

Existing primers from the literature [33] were evaluated using primer 3 tool to determine sequences for product amplification and sequencing.

### 2.11. Preparation of *In Vivo* Parasite DNA

A protocol outlined by Wooden et al. [36] was adopted with minimal changes. Each parasite DNA preparation was obtained from 50 *μ*l of PRBCs spotted on Whatman 903™ filter papers. DNA was extracted from the dried blood spots using Chelex method [36]. Parasitized red blood cells from the mice treated with CQ acted as the positive control. The concentration of DNA was calculated using a NanoDrop spectrophotometer. Finally, the DNA samples were stored at −20°C awaiting product amplification.

### 2.12. Amplification

A modified PCR programme was adopted [33]. Briefly, in a 25 *μ*l PCR, 1 *μ*l of genomic DNA per sample was used as template to successfully amplify *Pb*CRT and *Pb*MDR1 genes. The other components included dNTPs, MgCl_2_, forward and reverse primers, Dream Taq polymerase (Thermo Scientific), and correctly optimized cycling conditions as indicated in Table 1. Analysis of the PCR products was done in 1% agarose gel and purified using ExoSAP-IT® (Affymetrix, Santa Clara, CA) as directed by the manufacturer's protocol. The purified products were then sequenced as per Big Dye® Terminator v3.1 Cycle Sequencing Kit reaction mix (Applied Biosystems, USA) using 3500xL sequencer. The generated chromatograms from both the forward and reverse primers were trimmed using ChromasPro software (version 2.6.6) to remove poor reads. BioEdit software version 7.0.5.3 imported the edited reads. The reads underwent alignment and assembling into contigs after solving likely ambiguities by eye. MEGA-X software enabled evaluation of genetic polymorphisms in relation to the reference genes from PlasmoDB. The primers used for sequencing are captured in Tables 2 and 3.

#### 2.12.1. *Pb*MDR1 Amplification

The following primer set was used to amplify the whole coding region of *Pb*MDR1 gene:

 
*Pb*MDR 1F: ATCAGGAGCTTCGTTGCCTA
 
*Pb*MDR 9R: GGGCTTGAACAAAAGATCCA [33].

#### 2.12.2. *Pb*CRT Amplification

The primers for amplification of the whole coding region of the gene consisted of the following sequences: *Pb*CRT-1F: GGA CAG CCT AAT AAC CAA TGG *Pb*CRT-4R: GTT AAT TCT GCT TCG GAG TCA TTG [33].

The programme mentioned in Table 1 was adopted from Kangethe [33] with minor modification.

The *Pb*MDR1 sequencing primers provided in Table 2 were obtained from Kangethe [33].

The *Pb*CRT  sequencing primers provided in Table 3 were obtained from Kangethe [33].

### 2.13. Preliminary Qualitative Phytochemical Screening

Phytochemical studies for the two extracts were run as per the standard procedures described by Sheel et al. and Tiwari et al. [37, 38] with minimal changes as follows.

#### 2.13.1. Detection of Alkaloids

To 1 g of extract, dilute hydrochloric acid was added. Wagner's test: 4 drops of Wagner's reagent (iodine in potassium iodide) were added to the prepared sample. Development of brown/reddish precipitate showed the presence of alkaloids.

#### 2.13.2. Detection of Terpenoids

Salkowski test: 1 g of the extract was mixed with 2 ml of chloroform. 3 ml of concentrated sulphuric acid was cautiously added to form a layer. Formation of a reddish brown interface indicated the presence of terpenoids.

#### 2.13.3. Detection of Saponins

Foam test: 2 ml of water was added to 1 g of extract and shaken well. Persistence of the foam for 10 minutes showed the presence of saponins.

#### 2.13.4. Detection of Sterols

Salkowski's test: to 1 g of extract, 2 ml of chloroform and 2 ml of concentrated sulphuric acid were added and shaken well. Greenish yellow fluorescence at the red layer of chloroform and acid indicated the presence of sterols.

#### 2.13.5. Detection of Glycosides

1 ml of water was added to 1 g of extract and shaken well. 2 ml of aqueous solution of sodium hydroxide was then added. The appearance of yellow colour showed the presence of glycosides.

#### 2.13.6. Detection of Phenols

Ferric chloride test: 1 g of extract was treated with 4 drops of ferric chloride solution. Formation of bluish black colour indicated the presence of phenols.

#### 2.13.7. Detection of Tannins

Gelatin test: to 1 g of extract, 2 ml of 1% gelatin solution encompassing sodium chloride was added. Formation of white precipitate showed the presence of tannins.

#### 2.13.8. Detection of Flavonoids

Alkaline reagent test: 1 g of extract was treated with 4 drops of sodium hydroxide solution. Establishment of intense yellow colour, which turns colourless on addition of dilute hydrochloric acid, indicated the presence of flavonoids.

### 2.14. Statistical Analysis

*In vivo* assay analysis was done using Windows SPSS version 25. One-way analysis of variance (ANOVA) followed by Tukey's honest significant difference post hoc test was utilized to establish statistical significance for assessment of *in vivo* assay. A *P* value of less than 0.05 was considered statistically significant. The DNA sequences and the predicted amino acid sequences were analyzed by the MUSCLE algorithm using Mega-X software and searched by BLAST© software in NCBI website and PlasmoDB version 11.

### 2.15. Ethical Approval

The study received approval from KEMRI Scientific and Ethics Review Unit (SERU) under protocol number KEMRI/SERU/CBRD/191/3803. Every procedure in the study was carried out in accordance to KEMRI guidelines on animal care and use. Similarly, the WHO guiding principles on laboratory animal use and care were followed.

## 3. Results

### 3.1. Acute Toxicity Study

The extracts resulted in no mortality at a dose of up to 2000 mg/Kg within the first 24 h as well as successive 14 days. Moreover, there were no physical and behavioral signs of over-toxicity such as shrunk body or/and limb, reduced motor activity, abnormalities in breathing, struggling, dizziness, lack of appetite, rough fur, and spasms in both rear legs. This proposes that LD50 of the extracts is higher than 2000 mg/Kg.

### 3.2. *In Vivo* Antimalarial Activity of the Extracts against *P. berghei* ANKA

The antimalarial activity of the extracts against *P. berghei* ANKA is shown in Table 4. The % parasitemia is inversely proportional to % parasitemia suppression of the test groups. The parasitemia suppression activity is exhibited in a dose-dependent manner. At 500 mg/kg, the extracts revealed the highest parasite reduction activity. There was a significant percentage parasitemia difference between the test and the negative control groups (*P* < 0.001). However, there was a small difference in chemosuppression between the positive control and the two extracts. Interestingly, the extracts extended survival time of the animals after treatment closure (*P* < 0.001) as compared to the negative control.

### 3.3. Measurement of Packed Cell Volume

The packed cell volume (PCV) values were used to indicate anemia as a result of severity of malaria. The PCV values show a decrease in values, i.e., 49.80 ± 2.78 in the negative control as compared to test groups that were above 53 which indicates anemia. Therefore, the positive control and the extracts were able to reverse PCV reduction by preventing hemolysis arising from cumulative parasite manifestation. The data are captured in Table 5.

### 3.4. Resistance Index Values and ED50 and ED90 of Methanolic and Ethyl Acetate Extracts Measured in the 4-Day Test during the Drug Selection Pressure Assay

**Figure 1:**
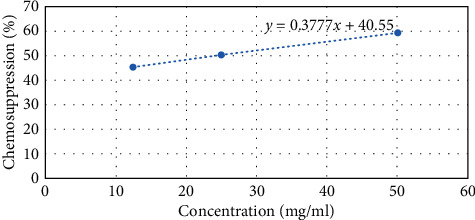
ED_50_ and ED_90_ at passage 16 (methanolic extract against *P. berghei* ANKA).

**Figure 2:**
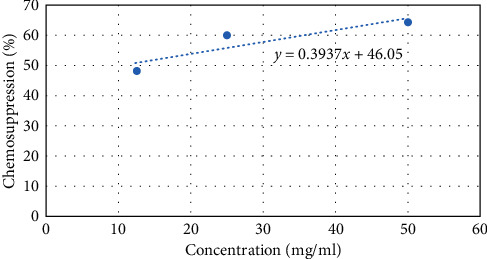
ED_50_ and ED_90_ at passage 16 (ethyl acetate extract against *P. berghei* ANKA).

At cycle 0, the ED_50_ for methanolic extract was 0.92 mg/ml and ED_90_ was 82.99 mg/ml. Notably, the ED_50_ and ED_90_s rose as the number of cycles increased. However, the resistance index values slightly rose in the 5^th^ passage at a slower rate. The RSI_50_ and RSI_90_ values of 2.30 and 1.01, respectively, demonstrate that, at passage 5, there is a slower resistance development. Particularly, at the 11^th^ passage, the ED_50_ and ED_90_ values are steadily increasing with RSI values of 6.8 and 1.32, respectively, illustrating slight resistance development. Interestingly, at passage 16, the ED_50_ and ED_90_ shot to 25.02 mg/ml and 130.92 mg/ml, respectively. The same trend is seen in the RSI values with RSI_50_ of 27.2 and RSI_90_ of 1.58. The escalated increase of RSI_50_ at passage 16 can indicate moderate resistance. Based on the ED_50_s, a 27-fold difference between the sensitive (cycle 0) and resistant lines (cycle 16) of methanolic extracts of *N. sativa* is recorded. At cycle 0, the ethyl acetate extract produced ED_50_ and ED_90_ values of 0.77 and 78.04 mg/ml. At passage 5, there was a slight increase of the ED_50_ and ED_90_ values with 2.67 and 79.10 mg/ml, respectively. However, there was steady increase of the ED_50_ and ED_90_ values with slight rise of RSI_50_ and RSI_90_ values of 6.35 and 1.15, respectively, showing slight resistance development. Significantly, at passage 16, the ED_50_ shot higher producing RSI_50_ of 13.03 and RSI_90_ of 1.43. Therefore, the value of RSI_50_ at cycle 16 indicates moderate resistance as it is higher than 10 but less than 100. Additionally, from the ED_50_s results, a 13-fold difference between the sensitive (cycle 0) and resistant lines (cycle 16) of ethyl acetate extracts of *N. sativa* is noted. The above-mentioned results are captured in Tables 6 and 7. Figures 1 and 2 provide a straight line of a dose-response curve of chemosuppression versus concentration of the methanolic and ethyl acetate extracts, respectively, demonstrating ED_50_ and ED_90_ at passage 16.

### 3.5. Sequencing Results

There were no polymorphic changes in the whole coding regions of the *Pb*MDR1 and *Pb*CRT genes. This was concluded upon comparison of the analyzed sequence genes with the reference genes.

### 3.6. Qualitative Phytochemical Investigation of the Five Extracts of *N. sativa*

We found out that the extracts had all the phytochemicals investigated except phenols in ethyl acetate extract. However, flavonoids, alkaloids, saponins, and terpenoids were strongly present. Tannins, sterols, and glycosides were weakly present. The outcomes of phytochemical profiling and respective extract yields are presented in Table 8.

## 4. Discussion

Numerous medicinal plants contain various compounds which serve as possible drug sources for human disease management [39]. The majority of the known antimalarials such as CQ, mefloquine, and recently artemisinin products have been discovered using rodent malaria model [8]. During rational drug discovery from plant sources, *in vitro* and/or *in vivo* assays are carried out. Generally, *in vivo* tests are more practical, more rapid, and less expensive than *in vitro* assays [40–42]. Molecular methods may also be incorporated as they are more accurate and provide a bigger picture of the future significance of the product under investigation [43,44].

A perfect antimalarial treatment exhibits selectivity and therapeutic activity on malaria parasites without consequential toxicity on the host. Acute toxicity investigation determined no death at a dose of up to 2000 mg/kg body weight in the first 24 h and the succeeding 14 days. Additionally, there was no record of bodily and behavioral signs that indicate over-toxicity. The investigated signs, including shrunk body or/and limb, reduced motor activity, abnormalities in breathing, struggling, dizziness, lack of appetite, rough fur, and spasms in both rear legs, as explained by Chen et al. [45] were not included in their study. Successful completion of the 4-day suppressive test with no resulting death explains the safety of the extracts. This is a clear indication that the lethal dose able to kill at least half of the experimental population (LD50) is higher than 2000 mg/kg of body weight. Elsewhere, Al-Sheddi et al. [46] determined that *N. sativa* at lower concentrations exhibited no cytotoxicity unlike higher concentrations. In essence, the effect of the plant on the animal host mirrors that of human. Therefore, the plant can easily be utilized in human host solely in reduced concentrations.

This study used *P. berghei* ANKA, an *in vivo* model which covers preclinical aspect in infection eradication for prediction of treatment outcomes. The *in vivo* evaluation showed significant dose-dependent reduction in percentage parasitemia on the test groups compared to the negative control group (*P* < 0.001). Consequently, at 500 mg/kg of the extracts, the parasite suppression hit 75.52 and 75.3% for methanolic and ethyl acetate, respectively. At lower dose of 125 mg/kg, the parasite suppression ability was 56.29 and 59.02% for methanolic and ethyl acetate, respectively. It appears that the active antimalarial compounds are mainly concentrated in higher doses. The parasite reduction capability is highest at the fourth day. This is due to high level of extract on day four due to continuous administration and, therefore, much is absorbed to the animal body system. This indicates that the chemical ingredients of the crude extracts are weakly or not affected by biotransformation, biodegradability, physiological factors, and bioavailability in the host. Considerably, survival time of the test animals was prolonged compared to the negative control animals. However, the survival time in the animals treated with CQ is much pronounced than the test animals. This could be due to the fact that CQ is a pure compound as compared to the crude extracts that are possibly metabolized easily from animal body. Elsewhere, Kangethe worked with artemisinin extracts and confirmed that the crude extracts find their way quickly out of the host bodies [33].

Secondary metabolites play a crucial role in drug establishment. Alkaloids, saponins, flavonoids, and tannins have recorded antimicrobial potency among a number of selected medicinal plants [47]. Our study identified the following: flavonoids, tannins, sterols, alkaloids, saponins, glucosides, and terpenoids except phenols. The phytochemical results are in conformity with those of Ishtiaq et al. [48] and Ashcroft et al. [21]. *Nigella sativa* has been demonstrated to exhibit antioxidant activity [21]. Monti et al. [49] explained the relevance of antioxidant activity to malaria parasite. Antioxidant activity prevents heme polymerization; hence, the toxic unpolymerized heme kills the malaria parasite. In a different study, alkaloids have been shown to confer antimalarial activity by hindering protein synthesis in *Plasmodia* parasites [50]. Moreover, saponins, flavonoids, and tannins act as free radical scavengers that counter the oxidative damage caused by the malaria parasites [51]. Hashem and El-Kiey [52] noted that tannins bond with proteins by hydrophobic forces preventing microbial enzyme activity and transport of protein. Flavonoids bond with nucleic acid bases suppressing *Plasmodia* cycle [53]. In a different study, a natural product thymoquinone, isolated from *N. sativa*, has been shown to possess antimalarial activity due to its antioxidant nature and has no adverse effects [54]. To the best of our understanding, the plant must have posted the reported antimalarial ability due to either a separate or combined phytochemical(s).

Anemia is one of the symptoms of malaria. Anemia levels are directly proportional to the severity of malaria. Human and murine model have a close linkage in anemia expression due to similarity in biological makeup [55]. Packed cell volume values are inversely proportional to anemia as a result of malaria [21]. There was an increase in PCV values in the test extracts ranging from 53.00 ± 1.58 to 53.60 ± 2.88 for the methanolic extract and 53.00 ± 1.58 to 53.60 ± 1.52 for the ethyl acetate extract. The results indicate increased PCV values in the test extracts when compared with the negative control but not statistically significant upon comparison with the positive control. This shows that the test extracts were able to endure hemolysis of the red blood cells that results in anemia. The study agrees with that of Ashcroft et al. [21] that *N. sativa* extracts demonstrate increased PCV values. Interestingly, Kaur et al. [55] found that saponins have a strong hemolytic effect which potentially reduces the PCV values. Our test extracts have been demonstrated to possess a strong presence of saponins in Table 8 which opens up a very interesting aspect to future investigation for the difference in our results and those of Kaur et al. [55].

Coquelin et al. [56] established that when parasites get their way into the red blood cells, they are susceptible to the drug. This could be the reason for the slight resistance development established at passage 5 of this study. The results of this study agree with previous study conducted by Xiao et al. [57] that continuous administration of constant dose at every passage cycle leads to resistance. At passage 16, we were able to determine moderate resistance. Still, from the drug pressure assay, stable resistant phenotype is exhibited as delayed parasite clearance from the microscopically detectable blood-phase infections on day 3 after treatment. It is expected that parasites die on exposure to an active drug contrary to the finding. Interestingly, young ring and older stages of parasites survived after 72 h of drug exposure. Consistent with this phenomenon, S. Ménard et al. [58] demonstrated artemisinin failing to clear parasites up to the 48th hour of drug exposure. They finally isolated a resistant phenotype in the form of delayed parasite clearance after 32 successive passages. Later, we noted no polymorphic change in position of the nucleotide sequences of the two genes: *Pb*MDR1 and *Pb*CRT. These results agree with the previous study run using lumefantrine and piperaquine resistant *P. berghei* ANKA that demonstrated no variation in the coding sequences in *Pb*CRT and *Pb*MDR1 genes [59]. Equally, Kangethe was unable to identify a recognizable mutation up to the 40th cycle of drug pressure *in vivo* [33]. The two genes influence drug activity by obtaining mutations and change in copy number and expression level in drug-resistant *P. falciparum* [60]. However, the resistance mechanism in *P. falciparum* and *P. berghei* may be diverse. We consequently reason out that the genetic mutation attainment *in vivo* is not entirely dependent on that of the *in vitro P. falciparum* due to difference in biological organization. Additionally, we propose that *P. berghei* ANKA response to *N. sativa is* not limited to the *Pb*MDR1 and *Pb*CRT genes. Therefore, unique ways may have influenced *Pb*MDR1 and *Pb*CRT genes not to establish a viable mutation.

## 5. Conclusion

*Nigella sativa* extracts show significant (*P* < 0.05) parasitemia reduction activity in all the antimalarial assessments. The toxicity study justifies the safety of the plant in treating malaria. The 4-day parasite suppression test drug pressure for prevalence of *Pb*mdr1 and *Pb*crt genes gives direction on future scientific utilization of the plant to determine mechanism of resistance. It is recommended that drug pressure should be continued for a longer period using pure phytochemical compounds of *N. sativa* to select high *N. sativa* resistant lines (RSI > 100). Stability study of the resistant line should also be carried out. Repeated incomplete treatment done by Nuralitha et al. [61] can also be adopted alongside continuous drug pressure to delay the emergence of drug resistance thus understanding new trends of the ever-changing *Plasmodium* genome. Future research should also implement stepwise increase of drug concentration during drug pressure. We finally justify the use of *N. sativa* plant traditionally in Mombasa folk medicine.

## Figures and Tables

**Table 1:** Summary of PCR programme.

PCR amplifying summaries	Temperature (°C)/time (min)	
	*Pb*CRT	*Pb*MDR1
Initial denaturation	95°C, 5 min	95°C, 5 min
Denaturation	95°C, 1 min	95°C, 1 min
Annealing temperature	49°C, 45 sec	48°C, 45 sec
Elongation	68°C, 6 min	68°C, 5 min
Final elongation	72°C, 10 min	72°C, 10 min
Primer (forward and reverse)	0.25 pmol/*μ*l each	0.25 pmol/*μ*l each
Mgcl2 (mM)	2.0	1.5
dNTPs (mM)	0.2	0.2
Cycles	35	35
Infinite hold	4°C	4°C

**Table 2:** *Pb*MDR1 sequencing primers.

Set 1	PB MDR 1F	ATCAGGAGCTTCGTTGCCTA
	PB MDR 1R	TCCATGCATAAACTTGAAATCG

Set 2	PB MDR 2F	CAATGTCGATAATTGAAGAAGCA
	PB MDR 2R	CCATACCAAAATCCCAAAGC

Set 3	PB MDR 3F	GCTTTGGGATTTTGGTATGG
	PB MDR 3R	TGTCGACAGCTGGTTTTCTG

Set 4	PB MDR 4F	TCGTCAAGTGGAAATGGTGA
	PB MDR 4R	TCTGCAATCTCTTTTTCTTTTCG

Set 5	PB MDR 5F	GCCCCTGGATTTTTATCGTC
	PB MDR 5R	AGCAAATGTTCGCGTTGTAA
Set 6	PB MDR 6F	TTACAACGCGAACATTTGCT
	PB MDR 6R	TTTTCTTCTATCCCCTTTACTGTCA

Set 7	PB MDR 7F	AGTTGGAGAAACTGGATGTGG
	PB MDR 7R	GATGTTGCATCACGCATTTC

Set 8	PB MDR 8F	TTCGGTAAACAAGATGCAACA
	PB MDR 8R	GGCTCTAGCAATAGCAACTCG

Set 9	PB MDR 9F	GGTGGTCAAAAACAACGAGT
	PB MDR 9R	GGGCTTGAACAAAAGATCCA

**Table 3:** *Pb*CRT sequencing primers.

Set 1	PbCRT 1F	GGA CAG CCT AAT AAC CAA TGG
	PbCRT 4R	CTGAAGTAACAAAACTATAATTTCCC

Set 2	PbCRT 2F	TCA GGA AGA AGT TGT GTC A
	PbCRT 2R	GAT AAG GAA AAA CTG CCA TC

Set 3	PbCRT 3F	GTG TTG GCA TGG TCA AAA TG
	PbCRT 3R	CTT GGT TTT CTT ACA GCA TCG

Set 4	PbCRT 4F	TGTTAGTTGTATACAAGGACCAGC
	PbCRT 4R	GTT AAT TCT GCT TCG GAG TCA TTG

**Table 4:** Mean of parasitemia, % parasitemia suppression, and survival time after treatment termination.

Drug/extract	Dose (mg/kg)	Mean ± SD parasitemia (%)	% suppression of parasite	Mean survival time (days)
MeOH	125	7.18 ± 0.73	56.29	10.50 ± 0.58
	250	5.58 ± 0.39	66.05	11.25 ± 0.96
	500	4.03 ± 0.67	75.52	16.25 ± 0.96

EtOAc	125	6.75 ± 0.31	59.02	11.00 ± 0.82
	250	4.72 ± 0.19	71.27	12.40 ± 1.14
	500	4.04 ± 0.87	75.3	15.60 ± 1.52

CQ		1.20 ± 0.23	92.59	28.25 ± 1.50

Vehicle		16.45 ± 3.08		5.00 ± 0.82

^*∗*^The results are expressed as mean ± SD.

**Table 5:** Packed cell volume on D0 and D4.

Animal group	Dose	PCV-D0	PCV-D4	% change
Control	0.2 vehicle	53.40 ± 2.30	49.80 ± 2.78	−6.74
	5 mg/kg CQ	52.40 ± 2.07	55.00 ± 1.87	4.96

MeOH	125 mg/kg	53.00 ± 1.00	53.00 ± 1.58	0.00
	250 mg/kg	51.75 ± 1.50	53.25 ± 0.50	2.90
	500 mg/kg	52.40 ± 1.82	53.60 ± 2.88	2.29

Control	0.2 ml vehicle	53.80 ± 2.78	49.80 ± 1.64	−7.43
	5 mg/kg CQ	53.80 ± 2.78	55.80 ± 1.92	3.72

EtOAc	125 mg/kg	53.20 ± 3.03	53.60 ± 1.52	0.75
	250 mg/kg	56.25 ± 3.86	56.75 ± 2.99	0.41
	500 mg/kg	52.40 ± 2.30	53.00 ± 1.58	1.15

^*∗*^The PCV is expressed as mean ± standard deviation.

**Table 6:** Summary of the ED50, ED90 (mg/ml.day), and resistance index values of methanolic extract measured in the 4-day test during the drug selection pressure assay.

Passage No	Methanolic extract			
	ED_50_	RSI	ED_90_	RSI
Parent line	0.92	1	82.99	1
5^th^	2.12	2.30	83.82	1.01
11^th^	6.26	6.8	109.33	1.32
16^th^	25.02	27.2	130.92	1.58

**Table 7:** Summary of the ED50, ED90 (mg/ml.day), and resistance index values of ethyl acetate extract measured in the 4-day test during the drug selection pressure assay.

Passage No	Ethyl acetate extract			
	ED_50_	RSI	ED_90_	RSI
Parent line	0.77	1	78.04	1
5^th^	2.67	3.47	79.10	1.01
11^th^	4.89	6.35	89.84	1.15
16^th^	10.03	13.03	111.63	1.43

**Table 8:** Qualitative phytochemical investigation of the five extracts of *N. sativa*.

Phytochemical	EtOAc	MeOH
Phenols	−	+
Flavonoids	++	++
Tannins	+	+
Sterols	+	+
Alkaloids	++	++
Saponins	++	++
Glycosides	+	+
Terpenoids	++	++
Extract yield (%)	3.00	2.75

^*∗*^(++) strongly present, (+) weakly present, and (−) absent. Extract yield is expressed as percentage (%).

## Data Availability

The data supporting the outcomes of this study are included within the article.

## Supplementary Materials

Supplementary Materials The supplementary materials encompass the *in vivo* and molecular experiments. Pictures of the Giemsa-stained slides to determine parasite growth and drug suppression have been provided. The smears stained blue/pink are demonstrated to be infected with malaria. Gel pictures of the two genes under investigation are also provided to show the correct bands of the genes.
