# Supplementary material for: Antimalarial Activity of Nigella sativa L. Seed Extracts and Selection of Resistance in Plasmodium berghei ANKA in a Mouse Model
Source: J Pathog. 2021 Feb 3;2021:6165950. doi: 10.1155/2021/6165950 (PMC7875626; doi:10.1155/2021/6165950)
Supplement: Supplementary Materials — The supplementary materials encompass the in vivo and molecular experiments. Pictures of the Giemsa-stained slides to determine parasite growth and drug suppression have been provided. The smears stained blue/pink are demonstrated to be infected with malaria. Gel pictures of the two genes under investigation are also provided to show the correct bands of the genes. [file 6165950.f1.pdf]

Photomicrographs of *in vivo* and molecular experiments

*Donor mice and subsequent parasitemia Giemsa stained photomicrograph

Mouse shivering out of malaria

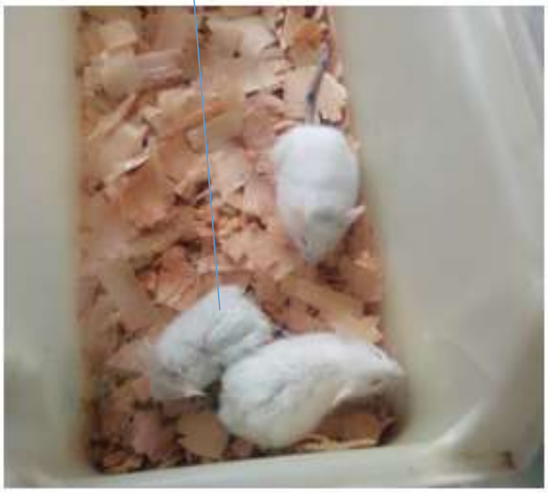

Red blood cell infected with *P. berghei* ANKA

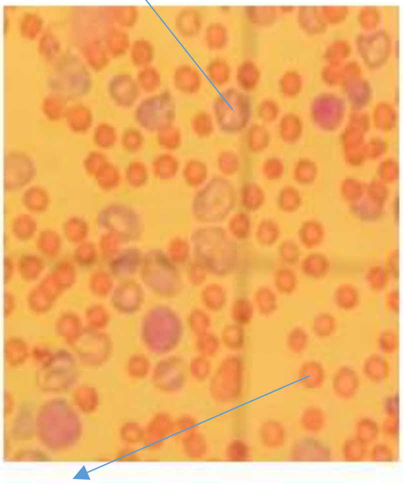

Normal red blood cell

* Parasite growth on day 1 in animals treated with Ethyl acetate extract

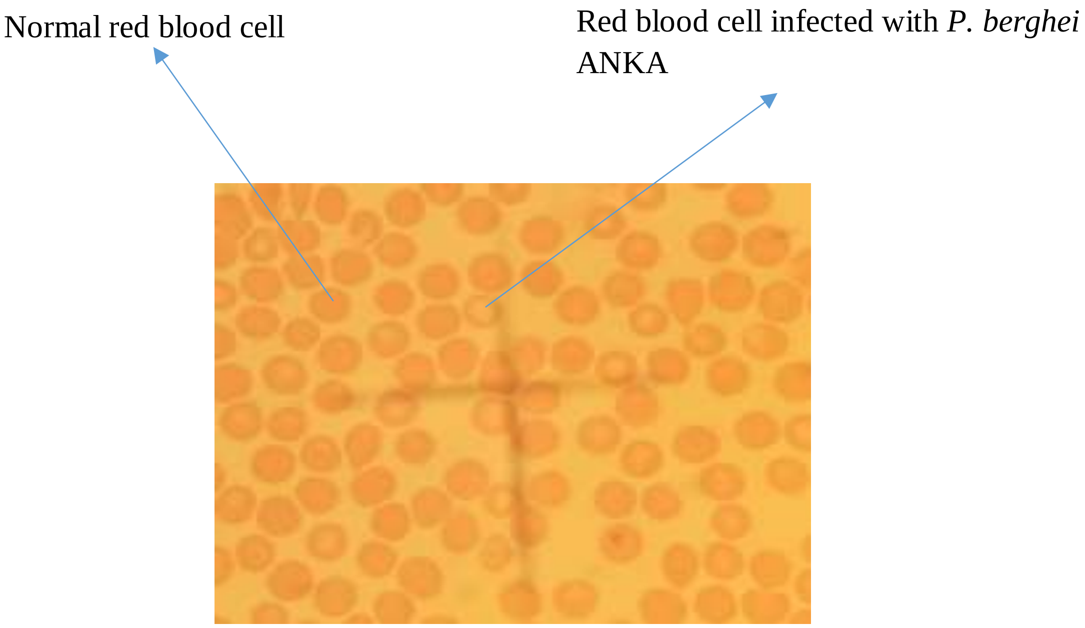

* Parasite growth on day 1 in animals treated with Methanol extract

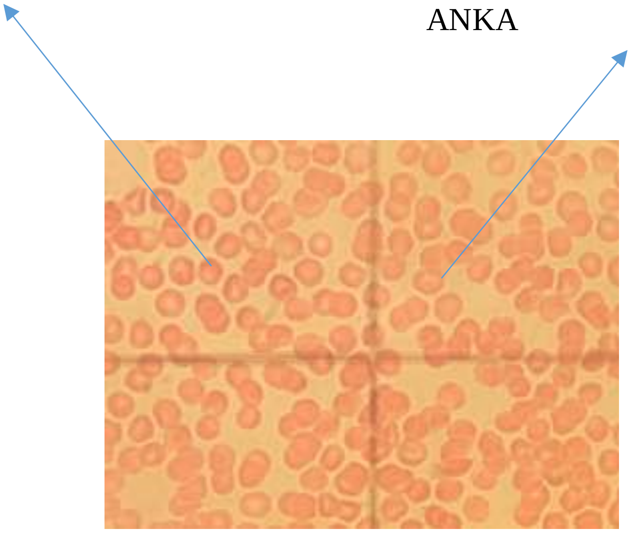

* Parasite clearance by Ethyl acetate extract

Normal red blood cell

Red blood cell infected with *P. berghei* ANKA

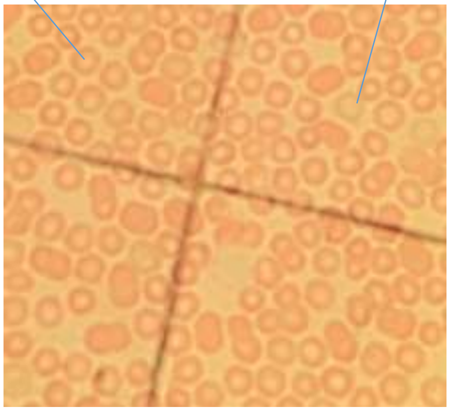

* Parasite clearance by Methanol extract

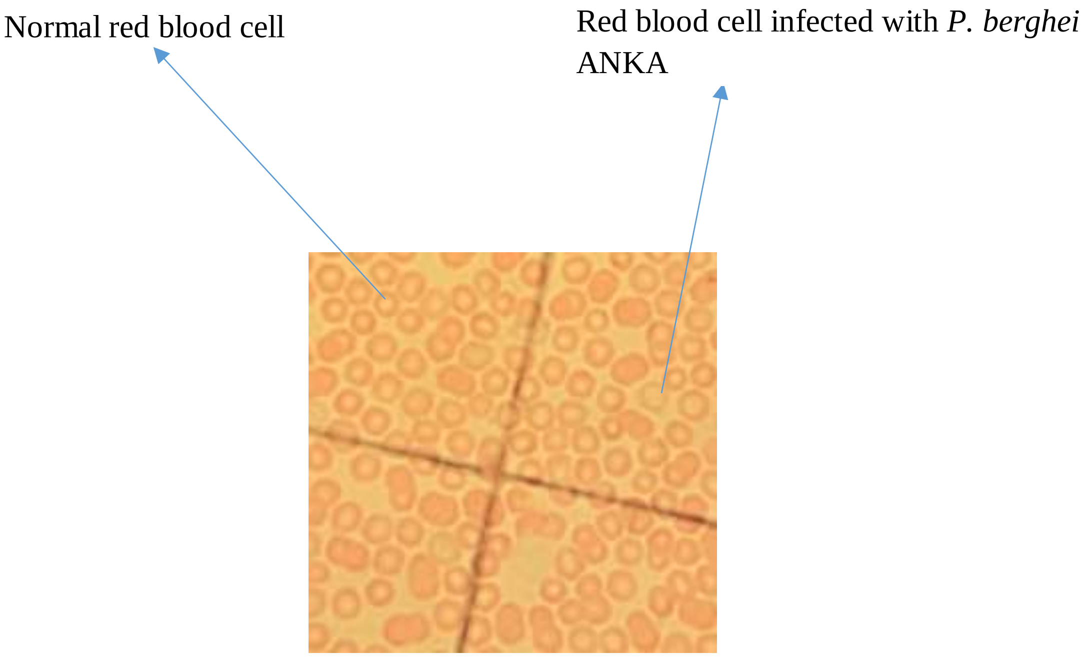

* Parasite clearance by Chloroquine

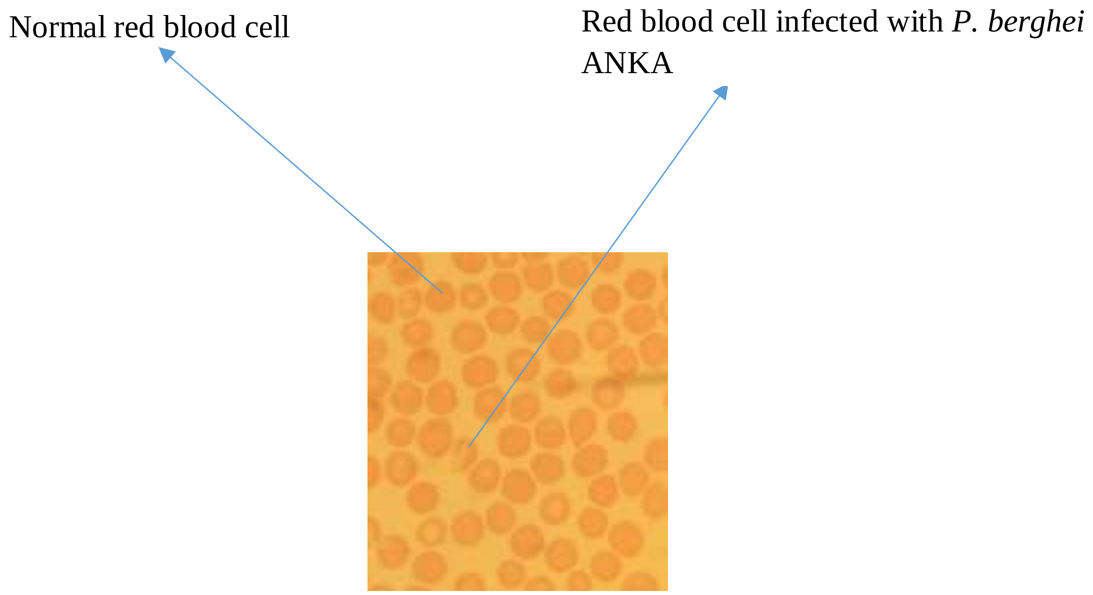

Amplification products with *Plasmodium berghei* ANKA

* PbMDR1 gel picture; 4002 bp

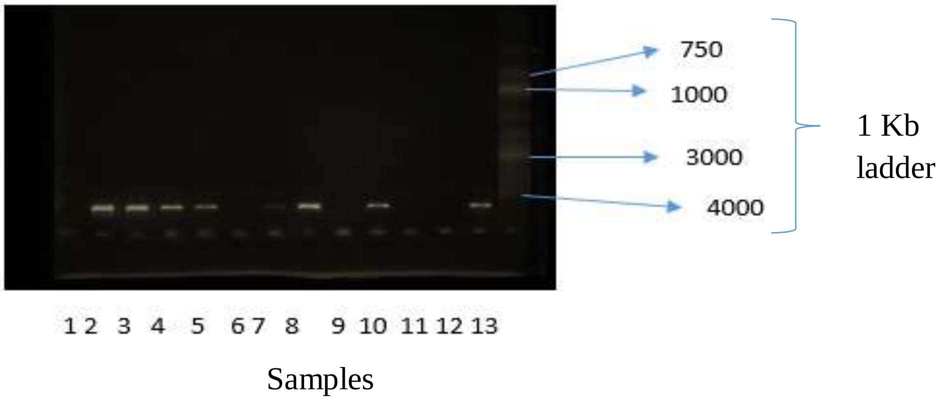

* PbCRT gel picture; 3077 bp

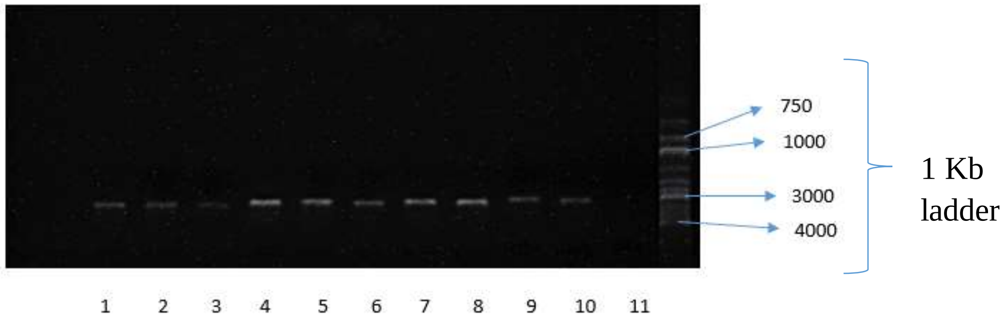

Samples

* Demonstration of liver infected with *P.berghei* ANKA versus normal liver

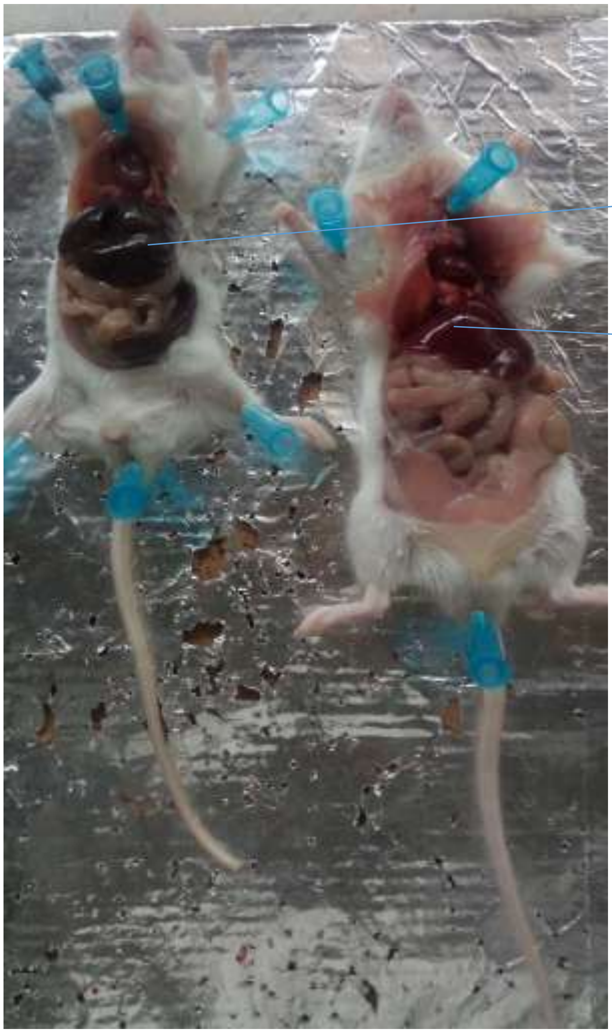

Liver infected with *P. berghei* ANKA

Normal liver
